# Clinical spectrum, outcome and impact of nephrectomy in critically-ill adult patients with emphysematous pyelonephritis: the PYELEMPHY observational retrospective multicenter Study

**DOI:** 10.1016/j.aicoj.2026.100086

**Published:** 2026-05-21

**Authors:** Keyvan Razazi, Pierre Louis Blot, Amélie Renou, Yannis Lombardi, Hugo Hille, Jérôme Devaquet, François Perier, Juliette Pocquet, Laurent Camous, Fréderic Pène, Sébastien Besset, Flora Delamaire, Anahita Rouze, Béatrice La Combe, Matthieu Petit, Laurent Laine, Jérémy Rosman, Romain Sonneville, Nicolas Mongardon, Alexy Tran Dinh, Stéphanie Houcke, Florence Boissier, Marc Pineton de Chambrun, Gregoire Jolly, Pierre Bailly, Pierrick Cronier, Malo Emery, Nahema Issa, Jean Loup Augy, Sébastien Jochmans, Jean Dellamonica, Claire Dupuis, Emmanuel Canet, Morgan Benais, Tomas Urbina, Antoine Goury, Damien Roux, Bastien Peiffer, Elsa Moncomble, Romain Arrestier, Igor Duquesne, Armand Mekontso Dessap, on behalf PYELEMPHY investigators

**Affiliations:** a AP-HP, Hôpitaux Universitaires Henri-Mondor, Service de Médecine Intensive-Réanimation, F-94010, Créteil, France; b Université Paris Est Créteil, Faculté de Médecine de Créteil, Institut Mondor de Recherche Biomédicale – Groupe de Recherche Clinique CARMAS, 94000 Créteil, France; c Université Paris Est Créteil, INSERM, IMRB, Créteil, F-94010, France; d Intensive Care Unit, Reunion University Hospital, Saint-Denis, France; e AP-HP Tenon, Service de Néphrologie, Paris, France; f Médecine Intensive Réanimation Centre Hospitalier Départemental de Vendée, La Roche-Sur-Yon, France; g Service de Réanimation Polyvalente, Hôpital Foch, 92150 Suresnes, France; h Réanimation Centre Hospitalier Versailles, Versailles, France; i Médecine Intensive-Réanimation, CHU Tours, Tours, France; j Service de Réanimation Centre Hospitalier Universitaire de Guadeloupe 97139 Les Abymes, France; k Service de Médecine Intensive-Réanimation, Hôpital Cochin, Assistance Publique-Hôpitaux de Paris (AP-HP), Centre-Université Paris Cité, 75014 Paris, France; l Service de Réanimation du Centre Hospitalier de Polynésie Française, Papeete, France; m Service de Réanimation Médicale, CHU Rennes, Rennes, France; n Univ. Lille, CNRS, Inserm, CHU Lille, U1285 - UMR 8576 -UGSF -Unité de Glycobiologie Structurale et Fonctionnelle, Service de Médecine Intensive Réanimation, F-59000 Lille, France; o Service de Réanimation Polyvalente, Groupe Hospitalier Bretagne Sud, Lorient, France; p Medical Intensive Care Unit, Ambroise Paré Hospital, Assistance Publique-Hôpitaux de Paris, Boulogne-Billancourt, France; q Médecine Intensive et Réanimation Hôpital Delafontaine, Centre Hospitalier de Saint Denis, Saint Denis, France; r Centre Hospitalier Intercommunal Nord-Ardennes, Site de Charleville-Mézières, Service de Réanimation, Unité de Recherche Clinique Ardennes Nord, Charleville-Mézières, France; s Medical and Infectious Diseases Intensive Care Unit (MI2), Bichat Hospital, AP-HP, Paris, France; t AP-HP, Hôpitaux Universitaires Henri Mondor, GRCT IMPACT, Service d’Anesthésie Réanimation, F-94010, Créteil, France; u Réanimation Chirurgicale, Bichat Hospital, AP-HP, Paris, France; v Service de Réanimation Polyvalente, Centre Hospitalier de Cayenne, Guyane Française, France; w Service de Médecine Intensive Réanimation, Centre Hospitalo-Universitaire de Poitiers, Poitiers, France; x Sorbonne Université, Assistance Publique-Hôpitaux de Paris (APHP), Hôpital La Pitié–Salpêtrière, Service de Médecine Intensive-Réanimation, Institut de Cardiologie, Paris, France; y CHU Rouen, Service de Médecine Intensive et Réanimation, F-76000, Rouen, France; z Service de Médecine Intensive et Réanimation, CHU de Brest, Brest, France; aa Service de Réanimation, CH Sud-Francilien, 40 Avenue Serge Dassault, 91100 Corbeil-Essonnes, France; ab Réanimation Polyvalente Hôpital Saint Camille, Bry-sur-Marne, France; ac Réanimation –Maladies Infectieuses Groupe Hospitalier Saint-André CHU de Bordeaux, Bordeaux, France; ad Service Médecine Intensive et Réanimation HEGP, APHP, Paris, France; ae Service de Médecine Intensive - Réanimation, GH Sud Ile-de-France, Hôpital de Melun-Sénart, Melun, France; af Service de Médecine Intensive – Réanimation, CHU de Nice UR2CA Université Cote d’Azur, Nice, France; ag CHU Clermont-Ferrand, Service de Réanimation Médicale, Clermont-Ferrand, Université Clermont Auvergne, Unité de Nutrition Humaine, INRAe, CRNH Auvergne, Clermont-Ferrand, France; ah Nantes Université, CHU Nantes, Médecine Intensive Réanimation, F-44000 Nantes, France; ai Réanimation Polyvalente, Centre Hospitalier de Valence, Valence, France; aj Service de Médecine Intensive Réanimation Hôpital Saint-Antoine, Sorbonne Université, Paris, France; ak CHU Reims, Médecine Intensive et Réanimation Polyvalente, F-51100 Reims, France; Université de Reims Champagne-Ardenne, Reims, France; al Médecine Intensive Réanimation, AP-HP, Hôpital Louis Mourier, DMU ESPRIT, 92700 Colombes, France; am Assistance Publique-Hôpitaux de Paris AP-HP, Hôpital Henri Mondor, Biostatistician, DMU Médecine, Créteil, France; an Assistance Publique-Hôpitaux de Paris AP-HP, Hôpital Henri Mondor, Service d’Urologie, Créteil, France

**Keywords:** ICU, Emphysematous pyelonephritis, Septic shock, Renal failure, Nephrectomy

## Abstract

**Background:**

Emphysematous pyelonephritis (EPN) is a rare, life-threatening necrotizing urinary tract infection marked by gas formation in the renal parenchyma and perirenal tissues. Evidence on its management and outcomes in the intensive care unit (ICU) remains limited. We aimed to describe the clinical profile, outcomes, and effect of nephrectomy in adults admitted to the ICU for infectious EPN.

**Main results:**

A national, multicenter, retrospective cohort study including adults admitted with infectious EPN to 48 French ICUs from 2001 to 2021 was conducted. A total of 109 patients were included. Median age was 62 years [IQR 54–72], and 64% were women. Overweight or obesity was present in 74%, and diabetes in 63%. Septic shock occurred in 72%. Urinary tract obstruction was identified in 46%. Initial management relied on antibiotics and/or minimally invasive drainage in 53% of cases. Early nephrectomy (≤24 h after ICU admission) was performed in 14 patients (14%), and delayed nephrectomy in eight (7%) at a median of 4 days [IQR 3–9]. Among the 75 patients discharged from the ICU without nephrectomy, four (4%) required subsequent nephrectomy. ICU mortality was 15% (n = 16). At day 90, vital status was known for 105 patients; 18 had died. In multivariable analysis, higher Simplified Acute Physiology Score II independently predicted increased 90-day mortality (HR per point 1.08; 95% CI 1.05–1.12; p < 0.001). Early nephrectomy was associated with lower mortality (HR 0.18; 95% CI 0.04–0.90; p = 0.037). Major adverse kidney events at day 90 occurred in 40%. Using the propensity-weighted model, early nephrectomy showed a trend toward lower MAKE90, although the association did not reach statistical significance (adjusted OR = 0.32; 95% CI: 0.10–1.02; p = 0.054).

**Conclusion:**

EPN requiring ICU admission carries substantial mortality. Early nephrectomy may improve survival, without increasing long-term renal complications.

## Highlights

• Emphysematous pyelonephritis is associated with high morbidity and mortality.
• Nephrectomy is frequently required.
• Early nephrectomy may improve survival.
• Nephrectomy was not associated with major adverse kidney events.

## Graphical abstract

**Figure:**
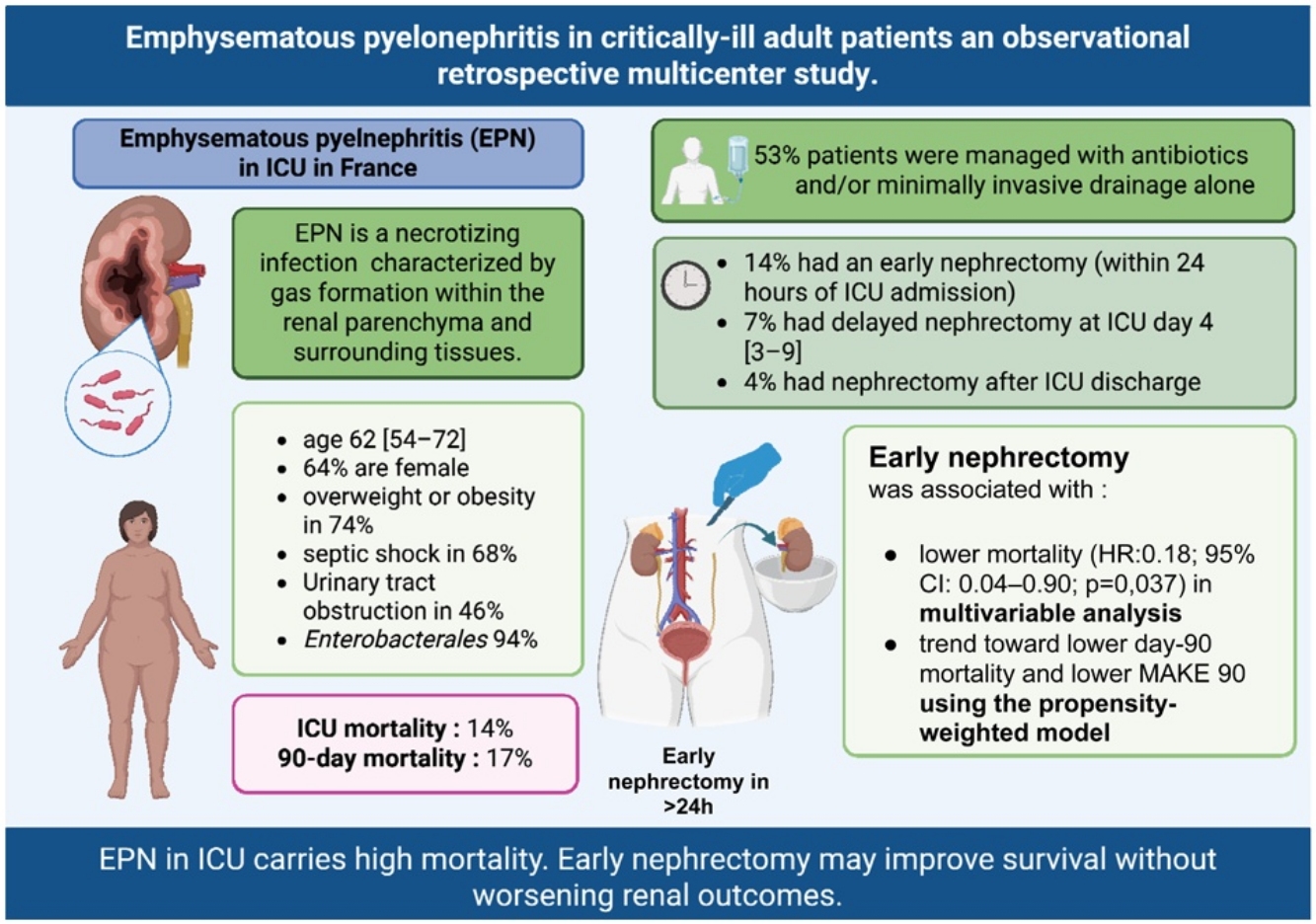

## Background

Emphysematous pyelonephritis (EPN) is a rare, life-threatening necrotizing infection of the upper urinary tract, characterized by the presence of gas within the renal parenchyma, collecting system, and/or perirenal tissues [1]. It predominantly affects adult women and is associated with substantial morbidity and mortality [[1], [2], [3]]. Diabetes mellitus and urinary tract obstruction are the main predisposing factors implicated in the pathogenesis of EPN [3]. Although the exact pathophysiological mechanisms are not fully elucidated, the most widely accepted hypothesis involves glucose fermentation by pathogenic bacteria, resulting in intrarenal gas production [4]. The clinical presentation is often nonspecific and potentially misleading, contributing to diagnostic delays and postponed initiation of appropriate management. Computed tomography (CT) is the imaging modality of choice, as it not only confirms the diagnosis by detecting gas within renal or perirenal structures but also provides essential prognostic information and guides therapeutic strategies [5].

EPN can lead to multiorgan failure and often requires admission to the intensive care unit (ICU). It also carries a high risk of renal dysfunction, either due to progressive renal necrosis or the need for nephrectomy, which may result in long-term kidney failure. Percutaneous drainage is now a key component of initial management [6]. However, the optimal treatment strategy in severe cases remains debated, particularly the role and timing of nephrectomy versus conservative management [[6], [7], [8]].

To date, no dedicated descriptive study has focused on critically ill patients with EPN, and the optimal management strategy in this setting remains uncertain. We therefore conducted a large multicenter retrospective cohort study of adult patients admitted to the ICU for infectious EPN. Our objective was to describe the clinical presentation, short- and long-term outcomes, and the impact of nephrectomy in this high-risk population.

## Methods

### Patients

All consecutive adult patients (≥18 years) admitted to the ICU for EPN between 2001 and 2021 across 48 ICUs in France (eTable S1, online supplement) were eligible for inclusion. In each participating center, a designated investigator was responsible for identifying eligible cases. Case identification was performed using the method deemed most appropriate by the local investigator. Keyword searches were conducted either in hospitalization report databases maintained by participating departments, for example using the Microsoft Windows® “search” function, or in institutional databases such as those of AP-HP, which allow anonymous keyword searches via “Cohort 360” to identify files containing the term “emphysematous pyelonephritis.” In addition, local investigators could identify cases using selected International Classification of Diseases, 10th Revision (ICD-10) codes within local hospital coding systems: N10 (acute tubulo-interstitial nephritis [pyelonephritis]), N28.0 (renal ischemia and infarction), N39.0 (urinary tract infection, site not specified), N15.1 (renal and perirenal abscess), and R57.2 (septic shock).

Clinical charts were reviewed by two investigators (KR and PLB) to confirm eligibility. EPN was defined by the presence of gas within the renal parenchyma, collecting system, or perirenal spaces, in the context of an acute urinary tract infection. Patients were excluded if they were moribund at admission, had postoperative fistulas with gas following nephrectomy, or had isolated emphysematous cystitis without renal involvement.

### Definitions

Early nephrectomy was defined as surgical removal of the affected kidney performed within the first 24 h following ICU admission. The extent of gas within the kidney and surrounding tissues was assessed on CT imaging by the local radiologist, using the Huang–Tseng classification system [5], as follows: Class 1, gas confined to the collecting system; Class 2, gas within the renal parenchyma without extrarenal extension; Class 3A, extension of gas or abscess into the perinephric space; Class 3B, extension beyond Gerota’s fascia; and Class 4, EPN involving both kidneys or a solitary functioning kidney.

Patient severity was assessed at ICU admission using the Simplified Acute Physiology Score (SAPS) II [9] and the Sequential Organ Failure Assessment (SOFA) score [10]. Acute Respiratory Distress Syndrome and septic shock were defined according to Berlin definition and Sepsis-3 criteria, respectively [11,12]. The Clavien–Dindo classification categorizes surgical complications into five grades: Grade I–II (minor complications requiring no or only pharmacologic treatment), Grade III (complications requiring surgical, endoscopic, or radiologic intervention), Grade IV (life-threatening complications requiring intensive care), and Grade V (death) [13]. Major adverse kidney events at day 90 (MAKE90) were defined as a composite outcome including death, the requirement for kidney replacement therapy, or a persistent reduction in renal function, indicated by a serum creatinine level ≥1.5 times baseline at day 90 [14,15]. When baseline serum creatinine was unavailable, values were estimated using the Chronic Kidney Disease–Epidemiology Collaboration (CKD-EPI) equation, assuming an estimated glomerular filtration rate (eGFR) of 75 mL/min/1.73 m^2^. MAKE90 was not assessed in patients who were already receiving chronic dialysis prior to ICU admission.

### Management

The clinical management of EPN was anchored on three pillars: supportive care, antimicrobial therapy, and source control. In the presence of urinary tract obstruction, source control systematically includes urinary drainage. The choice between percutaneous drainage and nephrectomy is based on the extent of emphysematous pyelonephritis on CT imaging, the severity of illness, and the patient’s clinical course.

### Statistical analysis

Categorical variables were expressed as number (%) and compared using the Chi-square or Fisher’s exact test, as appropriate. Continuous variables were reported as median [25th–75th interquartile range (IQR)] and compared using the Student’s t-test or Wilcoxon rank-sum test, depending on distribution. Survival analysis was censored at day 90, which was considered an appropriate timeframe to assess outcomes related to EPN management. Overall survival following EPN was defined as the time from ICU admission (or from the date of nephrectomy, to mitigate immortality bias) to death from any cause or the last available follow-up.

Early nephrectomy, SAPS II score assessed at ICU admission, and the CT-based classification of Huang and Tseng were included in the multivariable Cox proportional hazards regression model. To account for potential indication bias related to nephrectomy, additional multivariable analyses were conducted using overlap propensity score weighting. Variables included in the propensity score were Simplified Acute Physiology Score II and the Huang–Tseng classification. As the decision to perform nephrectomy was based on the extent of emphysematous pyelonephritis and the severity of the patient’s condition, these two clinically relevant variables, known to be associated with mortality, were included in the propensity score and the multivariable analysis. Standardized mean differences were examined to assess covariate balance between groups before and after weighting (eFig. S1). The analysis was repeated for MAKE90 using the same propensity score.

Statistical significance was defined as a two-tailed p value < 0.05. All analyses were performed using IBM SPSS Statistics version 22.0 (IBM Corp., Armonk, NY) and RStudio version 4.2.0 (https://www.R-project.org/).

### Ethical considerations

This retrospective observational study was approved by the Institutional Review Board of the French Intensive Care Medicine Society (CE SRLF 21-94, IRB No. 00014135). Data were collected anonymously from medical records and stored in a secure database declared to the National Commission on Information Technology and Civil Liberties (CNIL). The study methods and results are reported in accordance with the STROBE guidelines.

## Results

### Clinical features of adult patients with emphysematous pyelonephritis

**Fig. 1:**
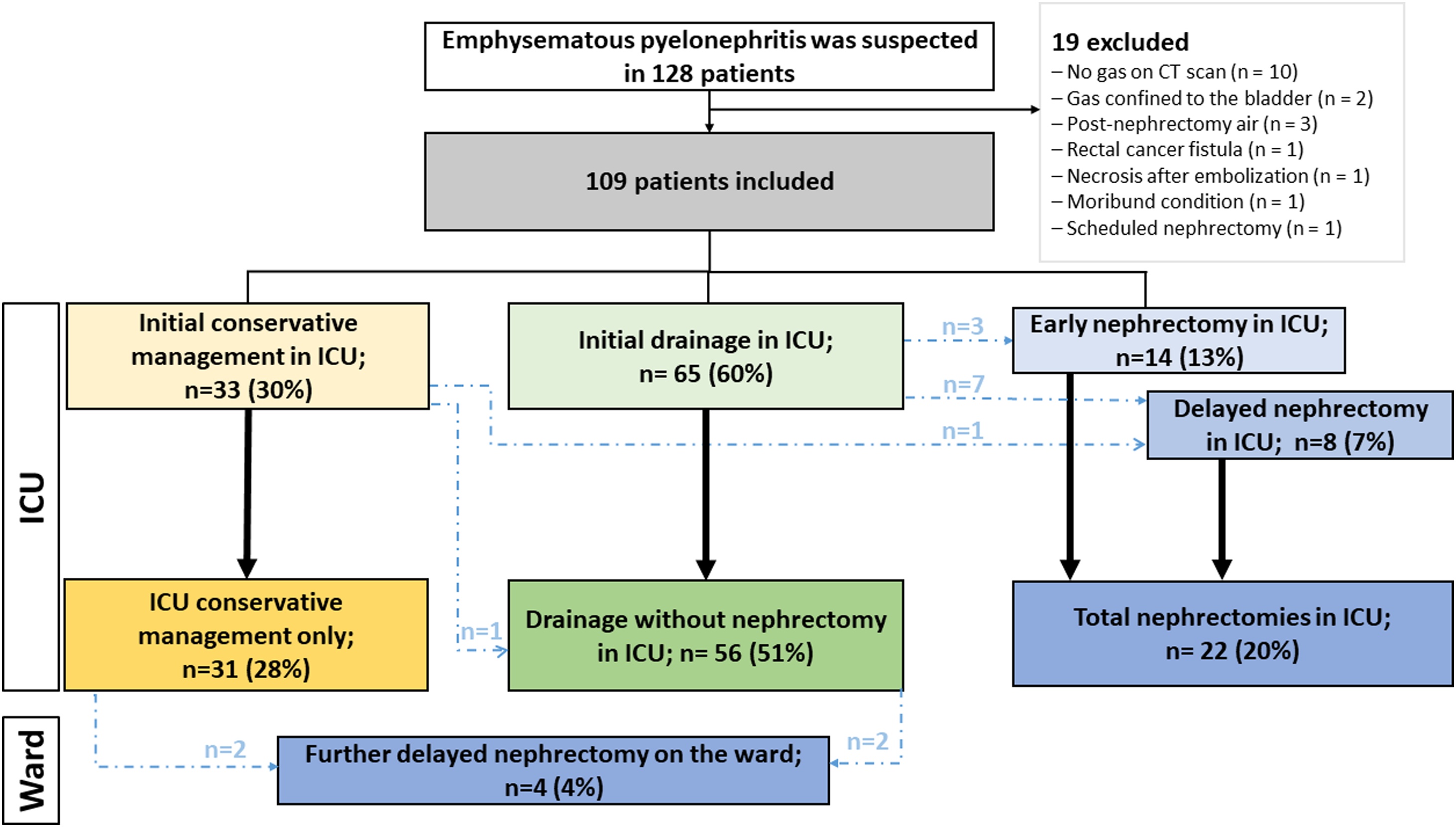
Flow chart and management strategies for emphysematous pyelonephritis (EPN).

Among 128 patients initially screened for suspected EPN, a total of 109 were included in the study, the majority of whom (n = 95; 91%) were admitted after 2010 (eTable S2, online supplement). Reasons for non-inclusion were as follows: absence of gas on CT scan (n = 10), gas limited to the bladder (n = 2), postoperative gas related to a recent nephrectomy (n = 3), renal necrosis following embolization for kidney cancer (n = 1), rectal cancer with a reno-rectal fistula (n = 1), moribund at admission (n = 1), and a scheduled nephrectomy (n = 1) (see flowchart in Fig. 1). Eleven participating centers reported no eligible cases during the study period. The distribution of included patients by center and by year is shown in eTable S1 and eTable S2 (online supplement). Patient characteristics and outcomes are summarized in Table 1. The median age was 62 years [54–71], with a female predominance (64%). A total of 63% of patients had diabetes, and 17% were classified as immunocompromised. Septic shock occurred in 72% of patients. Urinary tract obstruction was identified in 50 patients (46%). In most cases, ICU admission occurred within 24 hours of hospital admission. The most frequently isolated pathogen was *Escherichia coli* (n = 60), followed by *Klebsiella* spp. (n = 22), *Proteus mirabilis* (n = 6), *Enterococcus* spp. (n = 6), and other organisms (n = 5). Eight infections were polymicrobial.

**Table 1:** Demographic, clinical, biochemical, and radiological characteristics of the study population according to early nephrectomy.

	Available data		Early Nephrectomy in ICU		
Variables	N	[ALL] N = 109	No N = 95	Yes N = 14	P value
Female	109	70 (64%)	60 (63%)	10 (71%)	0.76
Age (years)	108	62 [54–71]	62.0 [54.2; 72]	56.0 [53; 63]	0.121
Medical history					
Diabetes mellitus	109	69 (63%)	61 (64%)	8 (57%)	0.830
Hypertension	109	62 (57%)	53 (56%)	9 (64%)	0.55
Alcohol abuse	101	24 (24%)	20 (22%)	4 (33%)	0.472
Body mass index (kg/m^2^)	59	26.8 [24.3; 30.8]	26.9 [24.1; 30.9]	26.0 [25.5; 27.0]	0.765
Chonic heart failure (NYHA 3–4)	109	15 (14%)	14 (15%)	1 (7%)	0.687
Chronic respiratory failure	109	3 (3%)	3 (3%)	0 (0%)	0,999
COPD	109	4 (4%)	4 (4%)	0 (0%)	0.999
Supraventricular arrhythmia	109	15 (14%)	13 (14%)	2 (14%)	0.999
Liver cirrhosis	109	10 (9%)	9 (9%)	1 (7%)	0.999
Cancer	109	11 (10%)	10 (11%)	1 (7%)	0.999
Hemopathy	109	4 (4%)	4 (4%)	0 (0%)	0.999
Immunodepression	109	19 (17%)	17 (18%)	2 (14%)	0.999
Chronic renal failure	109	24 (22%)	21 (22%)	3 (21%)	0.999
Urinary tract abnormality	109	29 (27%)	24 (25%)	5 (36%)	0.517
Recent NSAID	108	15 (14%)	12 (13%)	3 (21%)	0.408
Delay between hospital admission and ICU admission (days)	109	0.00 [0.00; 1.00]	0.00 [0.00; 1.00]	0.00 [0.00; 1.75]	0.716
Delay between diagnosis and early nephrectomy (days)	13			0 [0−1]	
Clinical characteristics upon ICU admission					
SAPS II at ICU admission	109	51 [37; 64]	49 [37; 63]	59 [51; 76]	0.055
SOFA score at ICU admission	102	8.00 [5.00; 12.0]	8 [4; 12]	12 [6; 15]	0.052
GCS at ICU admission	100	15 [14; 15]	15.0 [14.0; 15.0]	14.5 [12.0; 15.0]	0.259
Septic shock	109	78 (72%)	64 (67%)	14 (100%)	**0.009**
Bacteriemia	84	59 (70%)	50 (70%)	9 (69%)	0.999
Arterial blood lactate, mmol/L	94	3.50 [2.00; 5.70]	3.60 [2.10; 5.90]	2.90 [1.50; 5.00]	0.494
Norepinephrine, mg/h	92	1.50 [0.00; 4.12]	1.15 [0.00; 4.00]	3.00 [1.00; 10.3]	**0.047**
Mechanical ventilation	100	41 (41%)	31 (36%)	10 (77%)	**0.012**
White cell count, 10^9^/L	86	13.9 [9.05; 19.4]	13.9 [9.80; 19.0]	18.7 [7.60; 25.6]	0.933
Platelet count,10^9^/L	98	100 [53.2; 178]	102 [60; 178]	79.0 [20; 169]	0.198
Natremia, mmol/L	100	134 [128; 137]	133 [128; 137]	138 [124; 139]	0.300
Glycemia, mmol/L	82	10.7 [6.61; 18.7]	10.9 [6.61; 18.9]	7.85 [6.97; 17.1]	0.660
Total bilirubin, μmol/L)	94	13.0 [7.; 21.8]	12.1 [7.00; 20.0]	24.0 [12.0; 47.0]	**0.049**
Serum creatinine, μmol/L)	98	260 [152; 382]	257 [163; 357]	364 [141; 458]	0.408
Huang-Tseng scale	109				**0.005**
1−2		65 (60%)	62 (65%)	3 (21%)	
3−4		44 (40%)	33 (35%)	11 (79%)	
Huang-Tseng scale	109				0.016
1		34 (31%)	34 (36%)	0 (0%)	
2		31 (28%)	28 (30%)	3 (21%)	
3A		15 (14%)	11 (12%)	4 (29%)	
3B		17 (16%)	12 (13%)	5 (36%)	
4		12 (11%)	10 (11%)	2 (14%)	
Treatment at ICU admission					
Drainage	109	65 (60%)	62 (65%)	3 (21%)	**0.005**
Combinaison therapy with aminoglycoside	106	84 (79%)	71 (77%)	13 (93%)	0.291
Hydrocortisone	105	16 (15%)	14 (15%)	2 (15%)	0.999
Outcome and organ failure					
ARDS	105	18 (17%)	16 (18%)	2 (14%)	0.99
RRT in ICU	105	33 (31%)	25 (27%)	8 (62%)	**0.022**
ICU length of stay	109	5.00 [2.00; 11.0]	4.00 [2.00; 10.5]	8.00 [6.25; 13.0]	0.088
Death in ICU	109	16 (15%)	14 (15%)	2 (14%)	0.99
MAKE 90	87	35 (40%)	31 (41%)	4 (33%)	0.76
Death at day 90	105	18 (17%)	16 (18%)	2 (14%)	0.99
Dialysis at day 90*	87	5 (6%)	3 (4%)	2 (17%)	0.14
serum creatinine level ≥1.5 times baseline at day 90**	67	21 (31%)	19 (33%)	2 (22%)	0.71

ICU: intensive care unit; SAPS II: Simplified Acute Physiology Score II; SOFA: Sequential Organ Failure Assessment; GCS: Glasgow Coma Scale; NSAID: non-steroidal anti-inflammatory drug; COPD: chronic obstructive pulmonary disease; ARDS: acute respiratory distress syndrome, RRT: renal remplacement therapy.

* In patients alive at day 90 who were not receiving chronic dialysis before ICU admission.

** In patient alive and without dialysis at day 90. Baseline serum creatinine was estimated using the Chronic Kidney Disease–Epidemiology Collaboration (CKD-EPI) equation, assuming an estimated glomerular filtration rate (eGFR) of 75 mL/min/1.73 m^2^ in 25 patients.

### Management

Initial management consisted of conservative medical treatment in 33 patients (30%), minimally invasive drainage in 65 (60%), and early nephrectomy in 14 (13%), including three patients who underwent drainage followed by nephrectomy. During the ICU stay, one patient initially managed conservatively underwent delayed drainage, and another underwent delayed nephrectomy. Among those initially managed with drainage, seven patients required delayed nephrectomy in ICU. Following ICU discharge, two patients who had received conservative management in the ICU underwent delayed nephrectomy during their hospital stay, as did two patients managed with drainage in the ICU (Fig. 1).

In total, the 66 drainage procedures included 53 ureteral catheterizations (comprising 29 double-J stents and 24 standard ureteral catheters), seven percutaneous drainage, five percutaneous nephrostomies and one foley catheter placement in an ileal conduit. Delayed nephrectomies performed in the ICU and after ICU discharge occurred after a median delay of 4 [3–9] and 21 [20–25] days following ICU admission, respectively. Overall, eight of the 22 patients (36%) who underwent nephrectomy in the ICU experienced severe post operative complications (Clavien Dindo Grade ≥ 3). These included three cases of hemorrhagic shock, three pleural breaches, one renal fossa abscess, and one case of digestive perforation associated with iliac artery injury. The characteristics of the 14 patients who underwent early nephrectomy and their counterparts are summarized in Table 1. Patients in the early nephrectomy group more frequently met criteria for septic shock, had higher bilirubin levels and norepinephrine requirements and were more frequently classified as Huang–Tseng class 3 or 4. The characteristics of the 22 patients who underwent early nephrectomy in ICU and their counterparts are summarized in online supplement eTable S3.

### Outcomes

Sixteen patients (15%) died during their ICU stay. Patients who died in the ICU had more frequently cirrhosis as a comorbidity and were more severely ill at admission, as indicated by higher Simplified Acute Physiology Score II and Sequential Organ Failure Assessment scores. They also more often presented with bacteremia and met criteria for septic shock or Acute Respiratory Distress Syndrome. Furthermore, these patients had lower Glasgow Coma Scale values, higher arterial lactate levels, required higher doses of norepinephrine, and more frequently required combination therapy with aminoglycoside and mechanical ventilation. Urinary tract obstruction and the use of drainage procedures were not significantly different between ICU survivors and non-survivors (Table 2).

**Table 2:** Characteristics of patients according to ICU mortality (n = 109).

Variables	N Available data	Alive N = 93	Death N = 16	P value
Female	109	59 (63%)	11 (69%)	0.899
Age (years)	108	61.5 [54.0; 69.2]	62.0 [55.8; 76.2]	0.326
Medical history				
Diabetes mellitus	109	62 (67%)	7 (44%)	0.140
Hypertension	109	55 (59%)	7 (44%)	0.284
Alcohol abuse	101	21 (24.7%)	3 (19%)	0.756
BMI (kg/m^2^)	59	27.0 [23.6; 30.8]	25.8 [25.3; 27.5]	0.695
Chonic heart failure (NYHA 3−4)	109	15 (16%)	0 (0%)	0.121
Chronic respiratory failure	109	2 (2%)	1 (6%)	0.382
COPD	109	3 (3%)	1 (6%)	0.475
Supraventricular arrhythmia	109	14 (15%)	1 (6%)	0.693
Liver cirrhosis	109	5 (5%)	5 (31%)	**0.006**
Cancer	109	9 (10%)	2 (13%)	0.663
Hemopathy	109	4 (4%)	0 (0%)	1.000
Immunodepression	109	16 (17%)	3 (19%)	1.000
Chronic renal failure	109	20 (22%)	4 (25%)	0.749
Urinary tract abnormality	109	25 (27%)	4 (25%)	1.000
Recent NSAID	108	12 (13%)	3 (19%)	0.694
Delay between hospital admission and ICU admission (days)	109	0.00 [0.00; 1.00]	0.00 [0.00; 1.25]	0.830
Clinical characteristics upon ICU admission				
SAPS II at ICU admission	109	44.0 [36.0; 58.0]	78.5 [70.2; 87.2]	**<0.001**
SOFA score at ICU admission	102	8.00 [4.00; 11.0]	13.0 [12.0; 15.0]	**<0.001**
GCS at ICU admission	100	15.0 [14.0; 15.0]	12.0 [8.25; 14.8]	**0.002**
Septic shock	109	62 (67%)	16 (100%)	**0.005**
Bacteriemia	84	49 (66%)	10 (100%)	**0.029**
Arterial blood lactate, mmol/L	94	3.40 [1.80; 4.73]	9.05 [5.00; 13.2]	**<0.001**
Norepinephrine dose, mg/h	92	1.00 [0.00; 3.50]	12.0 [7.00; 16.5]	**<0.001**
Mechanical ventilation	100	31 (35%)	10 (83%)	**0.003**
White cell count, 10^9^/L	86	13.0 [8.90; 19.0]	14.3 [10.0; 28.0]	0.294
Platelet count,10^9^/L	98	102 [49; 176]	85.0 [58; 202]	0.958
Natremia, mmol/L	100	134 [128; 137]	132 [125; 136]	0.468
Glycemia, mmol/L	82	10.8 [6.60; 18.9]	9.30 [7.40; 14.7]	0.854
Total bilirubin, μmol/L	94	14 [7; 21]	13 [11; 26]	0.463
Serum creatinine, μmol/L	98	265 [144; 380]	242 [215; 368]	1.000
Huang-Tseng scale	109			0.093
1−2		59 (63%)	6 (38%)	
3−4		34 (37%)	10 (63%)	
Urinary tract obstruction	109	44 (47%)	6 (38%)	0.467
Treatment				
Any drainage	109	57 (61%)	8 (50%)	0.566
Combinaison therapy with aminoglycoside	106	69 (76%)	15 (100%)	**0.037**
Hydrocortisone	105	11 (12%)	5 (31%)	0.067
Nephrectomy in ICU	109	19 (20%)	3 (19%)	0.999
Outcome and organ failure				
ARDS	105	10 (11%)	8 (57%)	**<0.001**
RRT in ICU	105	26 (28%)	7 (54%)	0.106

ICU: intensive care unit; SAPS II: Simplified Acute Physiology Score II; SOFA: Sequential Organ Failure Assessment; GCS: Glasgow Coma Scale; NSAID: non-steroidal anti-inflammatory drug; COPD: chronic obstructive pulmonary disease; ARDS: acute respiratory distress syndrome; RRT: renal remplacement.

Vital status at day 90 was unavailable for four patients and they were therefore not included in the analysis; among the remaining, 18 (17%) had died by day 90 (eTable S4, online supplement). There was no significant difference in day-90 survival between patients who underwent early nephrectomy and those who received alternative management strategies in crude analysis (p = 0.78; see eFigure S2, online supplement). However, in multivariable analysis, early nephrectomy was independently associated with reduced day-90 mortality (HR = 0.18; 95% CI: 0.04–0.90; p = 0.037), whereas higher SAPS II scores were associated with increased mortality (HR per point = 1.08; 95% CI: 1.05–1.12; p < 0.001). These results were consistent in the overlap propensity score–weighted Cox model, which early nephrectomy remained significantly associated with reduced 90-day mortality (weighted HR = 0.17; 95% CI: 0.04–0.82; p = 0.027) (Table 3). In a propensity-weighted model, early nephrectomy showed a trend toward lower day-90 mortality, although the association did not reach statistical significance (adjusted OR = 0.26; 95% CI: 0.07–1.01; p = 0.051). The average treatment effect in the untreated (ATU) was −0.22 (95% CI: −0.39 to −0.005; p = 0.013).

**Table 3:** Univariate and multivariate analyses of variables associated with day-90 survival.

			Univariate analysis	Multivariable analysis	Mutivariable weighted analysis
Variables		Patients	HR (95% CI, p-value)	HR (95% CI, p-value)	wHR* (95% CI, p-value)
Early nephrectomy	No	95 (87.2)	–	–	–
					
	Yes	14 (12.8)	0.82 (0.19−3.54, p = 0.785)	**0.18 (0.04−0.90, p = 0.037)**	**0.17 (0.04−0.82, p = 0.027)**
SAPS II (per point)			1.07 (1.05−1.10, p < 0.001)	**1.08 (1.05−1.12, p < 0.001)**	**1.09 (1.06−1.12, p < 0.001)**
Huang–Tseng classification	1−2	65 (59.6)	–	–	–
				
	3−4	44 (40.4)	1.89 (0.75−4.80, p = 0.178)	2.37 (0.89−6.33, p = 0.084)	2.97 (0.96−9.13), p = 0.058

* Weighted Hazard Ratio (weights from the overlap weighting method).

MAKE90 status was available for 87 patients, of whom 35 (40%) met the criteria for major adverse kidney events (Table 1 and eTable S3). There was no significant association between early nephrectomy and the occurrence of MAKE90 (4/12 [33%] vs. 31/75 [31%], p = 0.76). Similarly, nephrectomy during ICU stay (8/19 [42%] vs. 27/68 [40%], p = 0.85) and nephrectomy at any time during hospitalization (9/23 [39%] vs. 26/64 [41%], p = 0.90) were not associated with an increased risk of major adverse kidney events at day 90. Using the propensity-weighted model, early nephrectomy showed a trend toward lower MAKE90, although the association did not reach statistical significance (adjusted OR = 0.32; 95% CI: 0.10–1.02; p = 0.054).

## Discussion

We report here a large multicenter cohort of adult patients admitted to the ICU for EPN. The main findings are as follows: (1) most patients were managed with antibiotics alone or with minimally invasive procedures, while nephrectomy was performed in one out of five patients; (2) the SAPS II was independently associated with mortality, whereas early nephrectomy was associated with a lower day-90 mortality in both multivariable analysis and overlap propensity score–weighted Cox model. In propensity-weighted models, early nephrectomy was associated with a trend toward lower day-90 mortality and reduced major adverse kidney events at day 90, although these associations did not reach statistical significance.

Previous studies on EPN have often included a high proportion of patients from developing countries, encompassing both ward and ICU populations. In these cohorts, patients with sepsis or septic shock were either poorly defined or underrepresented. Reported mortality ranged from 2% to 40%, reflecting considerable heterogeneity in disease severity [16]. Several clinical and biological factors (such as shock, hyperleukocytosis, thrombocytopenia, anemia, coma, and gas extension) have been identified as predictors of mortality or the need for ICU admission in EPN [16]. However, our study is the first to specifically examine risk factors for mortality in patients admitted to the ICU for EPN. A recent study [17] and several meta-analyses [16] have reported higher mortality in patients undergoing nephrectomy, whether performed emergently or after a delay. This association likely reflects confounding by indication, as nephrectomy is more often performed in the most severe cases, as those with extensive gas spread and septic shock. To assess the potential benefit of early nephrectomy, which remains a critical decision for intensivists upon ICU admission, we developed a propensity score accounting for gas extension and illness severity. In adjusted analyses, early nephrectomy was independently associated with improved ICU and day-90 survival. Histopathological findings in these patients frequently revealed impaired tissue perfusion, with abscess formation, microinfarctions or large infarcts, and vascular thrombosis [5], which may explain the limited efficacy of antibiotics alone. These observations support potential role of nephrectomy as an effective source control strategy in ICU patients with EPN. The approach to source control in EPN should be individualized. If obstruction is present, prompt decompression via ureteral stenting or nephrostomy is critical to facilitate drainage and reduce intrarenal pressure. Patients with Huang-Tseng class 1–2 or 3A, or with abscess formation usually respond well to percutaneous drainage. Partial nephrectomy and surgical lavage of the renal fossa can sometimes be used as source control strategies in emphysematous pyelonephritis, but are uncommon and generally reserved for select cases with localized disease, while total nephrectomy remains the standard surgical approach for extensive or refractory infection.

Data on MAKE and long-term outcomes in patients with EPN remain scarce in the current literature. MAKE is defined as a composite endpoint encompassing death, dialysis dependence, and persistent renal dysfunction, all of which are patient-centered outcomes. It has been proposed as a relevant measure in clinical studies evaluating renal failure [14,18]. In our cohort, the rate of MAKE was similar to that reported in a large study of sepsis-associated acute kidney injury, which found a 37% incidence of MAKE at hospital discharge [19]. Interestingly, nephrectomy was not associated with an increased risk of MAKE at day 90. In both living kidney donors and patients undergoing unilateral nephrectomy, the remaining kidney typically undergoes compensatory hypertrophy and hyperfiltration, restoring a major part of the baseline glomerular filtration rate within weeks to months [20]. The annual rate of renal function decline after nephrectomy is generally low, but the presence of pre-existing CKD, postoperative acute kidney injury, and albuminuria further increase the likelihood of long-term renal insufficiency after nephrectomy. These patients therefore warrant nephrology follow-up. Long-term studies are warranted to evaluate renal outcomes over years in this specific population.

Our study has several limitations. First, its retrospective design is associated with inherent biases, including missing data, particularly for day-90 follow-up. Second, the generalizability of our findings to patients managed in developing countries is uncertain, as the study population was exclusively drawn from French ICUs. In addition, because treatment decisions, including conservative management or interventional procedures, were made by local clinicians, variability in practice patterns may have introduced additional bias and influenced the observed outcomes. Third, the relatively small number of patients who underwent early nephrectomy warrants cautious interpretation of the associated outcomes, as not all potential confounding factors may have been accounted for, and the use of propensity-weighting and multivariable logistic regression models in a small sample should also be interpreted cautiously. Fourth, as the Huang–Tseng classification was assessed by a single local radiologist, misclassification of disease severity cannot be excluded. Finally, the long study period may have introduced variability in clinical practices, potentially affecting the consistency of management strategies. However, it is worth noting that the majority of patients were admitted after 2010, which reflects the limited availability of complete medical records from earlier years and the possibility of incomplete case identification during the case retrieval process cannot be excluded.

## Conclusion

EPN is associated with high morbidity and mortality. Nephrectomy was not infrequently required and may be considered in the most severely ill patients to ensure adequate source control.

## Authors’ contributions

Dr Razazi and Dr Blot had full access to all of the data in the study and takes responsibility for the integrity of the data and the accuracy of the data analysis.

Concept and design: Razazi, Mekontso Dessap.

Acquisition, analysis, or interpretation of data: All authors.

Drafting of the manuscript: Razazi, Mekontso Dessap.

Critical revision of the manuscript for important intellectual content: All authors.

Statistical analysis: Peiffer.

## Consent for publication

Not applicable.

## Ethics approval and consent to participate

This retrospective observational study was approved by the Institutional Review Board of the French Intensive Care Medicine Society (CE SRLF 21-94, IRB No. 00014135). Patients were informed of their inclusion in the study and written consent was waived in accordance with French law.

## Funding

None.

Group Information: The PYELEMPHY study group Investigators.

## Availability of data and materials

the datasets generated and/or analyzed during the current study are available from the corresponding author upon reasonable request.

## Declaration of competing interest

A.M.D. reports grants and personal fees from Fisher & Paykel, Baxter, Air Liquide, and Addmedica, all outside the submitted work.

K.R. received lecture fees from MSD and Shionogi, as well as a travel grant from Pfizer, all outside the submitted work.

EC has received lecturer and conference-speaker fees, as well as reimbursements of traveland accommodation expenses related to attending scientific meetings, from Gilead, Shionogi, and Sanofi-Genzyme.

No other disclosures were reported.
