# Supplementary material for: Clinical spectrum, outcome and impact of nephrectomy in critically-ill adult patients with emphysematous pyelonephritis: the PYELEMPHY observational retrospective multicenter Study
Source: Ann Intensive Care. 2026 May 21;16:100086. doi: 10.1016/j.aicoj.2026.100086 (PMC13218113; doi:10.1016/j.aicoj.2026.100086)
Supplement: Supplementary file 1 [file mmc1.docx]

**Clinical spectrum, outcome and impact of nephrectomy in critically-ill adult patients with emphysematous pyelonephritis: The PYELEMPHY observational retrospective multicenter Study (online supplement)**

Keyvan Razazi MD, PhD^1,2,3*^; Pierre Louis Blot MD^1*^; Amélie Renou MD^4^; Yannis Lombardi MD *^5^*; Hugo Hille MD*^6^*; Jérôme Devaquet MD ^7^; François Perier MD^8^; Juliette Pocquet MD^9^; Laurent Camous MD ^10^; Fréderic Pène MD, PhD^11^; Sébastien Besset MD^12^; Flora Delamaire MD^13^; Anahita Rouze MD^14^; Béatrice La Combe MD, PhD^15^ ; Matthieu Petit MD^16^; Laurent Laine MD^17^; Jérémy Rosman MD^18^; Romain Sonneville MD, PhD^19^; Nicolas Mongardon MD, PhD^20^; Alexy Tran Dinh MD, PhD^21^; Stéphanie Houcke MD^22^; Florence Boissier MD, PhD^23^ Marc Pineton de Chambrun MD, PhD^24^ Gregoire Jolly MD^25^ ; Pierre Bailly MD^26^; Pierrick Cronier MD^27^ ; Malo Emery MD^28^ ; Nahema Issa MD^29^; Jean Loup Augy MD^30^; Sébastien Jochmans MD^31^; Jean Dellamonica MD, PhD^32^; Claire Dupuis MD, PhD^33^; Emmanuel Canet MD, PhD^34^; Morgan Benais MD^35^, Tomas Urbina MD^36^; Antoine Goury MD^37^; Damien Roux MD, PhD^38^; Bastien Peiffer ^39^, Elsa Moncomble MD^1,2^; Romain Arrestier MD^1,2^, Igor Duquesne MD ^40^; Armand Mekontso Dessap MD, PhD^1,2,3,^ on behalf PYELEMPHY investigators $

**eTable 1. Number of Patients Included per Participating Center**

| **Centre** | **Number** | **Authors** |
| --- | --- | --- |
| Medical intensive care unit, Ambroise Paré Hospital, assistance Publique-hôpitaux de Paris, Boulogne-Billancourt, France | 3 | Matthieu Petit |
| Réanimation chirurgicale, Bichat Hospital, AP-HP, Paris, France. | 2 | Alexy Tran Dinh |
| Medical and Infectious Diseases Intensive Care Unit (MI2), Bichat Hospital, AP-HP, Paris, France. | 2 | Romain Sonneville |
| Réanimation –Maladies infectieuses Groupe hospitalier Saint-André CHU de Bordeaux, Bordeaux, France | 1 | Nahema Issa |
| Service de médecine intensive et réanimation, CHU de Brest , Brest, France | 2 | Pierre Bailly |
| Service de Réanimation Polyvalente, Centre Hospitalier de Cayenne, Guyane Française | 2 | Stéphanie Houcke |
| Centre Hospitalier Intercommunal nord-Ardennes, site de Charleville-Mézières, Service de Réanimation, Unité de Recherche Clinique Ardennes Nord. | 3 | Jérémy Rosman |
| CH Sud-Francilien, 40 avenue Serge Dassault, 91100 Corbeil-Essonnes, France | 2 | Pierrick Cronier |
| CHU Clermont-Ferrand, Service de Réanimation Médicale, Clermont-Ferrand, Université Clermont Auvergne, Unité de Nutrition Humaine, INRAe, CRNH Auvergne, Clermont-Ferrand, France | 1 | Claire Dupuis |
| Service de Médecine Intensive-Réanimation, Hôpital Cochin, Assistance Publique-Hôpitaux de Paris (AP-HP), | 4 | Frederic Pene |
| Thoracic Intensive Care Unit, Hôpital Foch, 92150 Suresnes, France | 6 | Jérome Devaquet |
| Service médecine intensive et réanimation HEGP APHP, Paris France | 1 | Jean Loup Augy |
| Médecine Intensive Réanimation Centre Hospitalier Départemental de Vendée, La Roche-Sur-Yon, France | 6 | Hugo Hille |
| Médecine Intensive Réanimation, AP-HP, Hôpital Louis Mourier, DMU ESPRIT, 92700 Colombes, France | 1 | Damien Roux |
| Department of Intensive Care Medicine, Critical Care Centre, CHU Lille, F-59000 Lille, France. | 4 | Anahita Rouze |
| Service de Réanimation Polyvalente, Centre Hospitalier Bretagne Sud, 56100 Lorient, France | 4 | Béatrice Lacombe |
| Service de Médecine Intensive - Réanimation, GH Sud Ile-de-France, Hôpital de Melun-Sénart, Melun, France | 1 | Sébastien Jochmans |
| Réanimation Centre hospitalier Versailles, Versailles, France | 6 | Francois Perier |
| AP-HP, Hôpitaux Universitaires Henri-Mondor, Service d’Anesthésie réanimation, F-94010, Créteil, France Service d’A-R chirurgicale, CHU Henri Mondor Créteil, France | 2 | Nicolas Mongardon |
| AP-HP, Hôpitaux Universitaires Henri-Mondor, Service de Médecine Intensive-Réanimation | 5 | Pierre Louis Blot |
| Nantes Université, CHU Nantes, Médecine Intensive Réanimation, F-44000 Nantes, France. | 1 | Emmanuel Canet |
| Service de Médecine Intensive – Réanimation, CHU de Nice UR2CA Université Cote d’azur, Nice, France | 1 | Jean Dellamonica |
| Service de réanimation du centre hospitalier de Polynésie française, Papeete, France | 4 | Sébastien Besset |
| Sorbonne Université, Assistance Publique-Hôpitaux de Paris (APHP), Hôpital La Pitié–Salpêtrière, Service de Médecine Intensive-Réanimation, Paris, France. | 2 | Marc Pineton de Chambrun |
| Service de réanimation Centre Hospitalier Universitaire de Guadeloupe 97139 Les Abymes, France | 5 | Laurent Camous |
| Service de Médecine Intensive Réanimation, Centre Hospitalo-Universitaire de Poitiers, Poitiers, France | 2 | Florence Boissier |
| CHU Reims, Médecine Intensive et Réanimation Polyvalente, F-51100 Reims, France; Université de Reims Champagne-Ardenne, Reims, France. | 1 | Antoine Goury |
| Service de Réanimation Médicale, CHU Rennes, Rennes, France | 4 | Flora Delamaire |
| Intensive Care Unit, Reunion University Hospital, Saint-Denis, France. | 9 | Amélie Renou |
| CHU Rouen, service de médecine intensive et réanimation, F-76000, Rouen, France. | 2 | Gregoire Jolly |
| Service de Médecine Intensive Réanimation Hôpital Saint-Antoine, paris, France | 1 | Tomas Urbina |
| Réanimation polyvalente hôpital saint Camille, Bry-sur-Marne, France | 1 | Malo Emery |
| Médecine intensive et réanimation Hôpital Delafontaine, Centre hospitalier de Saint Denis, France | 3 | Laurent Laine |
| APHP Tenon, service de néphrologie, Paris, France. | 7 | Yannis Lombardi |
| Tenon réanimation | 0 | Guillaume Voiriot |
| Médecine Intensive-Réanimation, CHU Tours, Tours, France | 6 | Juliette Pocquet |
| Réanimation polyvalente, Centre Hospitalier de Valence, Valence, France. | 1 | Morgan Benais |
| Réanimation Polyvalente, Centre Hospitalier Victor Dupouy Argenteuil, France | 0 | Damien Contou |
| Service de Médecine Intensive et Réanimation Hôpital Saint Louis (AP-HP), Paris , France | 0 | Virginie Lemiale |
| Département de Néphrologie et Transplantation d’organes, Centre Hospitalier Universitaire de Toulouse, France | 0 | Stanislas Faguer |
| Réanimation et Soins continus, Centre hospitalier Roanne. Roanne, France | 0 | Pascal Beuret |
| Service de Médecine intensive - Réanimation Département R3S Groupe Hospitalier Universitaire APHP-Sorbonne Université | 0 | Maxens Decavèle |
| Service de Médecine Intensive Réanimation, Grand Hôpital de l’Est Francilien Marne la Vallée, Marne la Vallée, France | 0 | Frank Chemouni |
| Médecine Intensive Réanimation, Hôpital Raymond Poincaré, Assistance Publique, Hôpitaux de Paris (AP-HP), Garches, France | 0 | Nicholas Heming |
| Service de Médecine Intensive et Réanimation Hôpital de Cannes Simon Veil, Cannes, France | 0 | Alexandre Robert |
| Service de Réanimation Polyvalente et Surveillance Continue, AP-HP, Hôpital Antoine Béclère, Clamart, France | 0 | Benjamin Sztrymf |
| Réanimation et surveillance continue adulte, Centre Hospitalier Intercommunal, , Créteil, France | 0 | Jérôme Cecchini |
| Service de Réanimation Polyvalente, Centre Hospitalier Saint Joseph-Saint Luc, Lyon, France | 0 | Emmanuel Vivier |

**eTable 2. Number of Patients Included per Year (2001–2021)**

| **year** | **Number of patients included** |
| --- | --- |
| 2001-2005 | 3 |
| 2006-2010 | 11 |
| 2011-2015 | 32 |
| 2016-2020 | 44 |
| 2021-2022 | 19 |

**eTable 3. Demographic, Clinical, Biochemical, and Radiological Characteristics of the Study Population According to Nephrectomy Status**

| **Variables** | **Available data** |  | **Nephrectomy in ICU** | | **P value** |
| --- | --- | --- | --- | --- | --- |
|  | **N** | **[ALL] N=109** | **No N=87** | **Yes N=22** |  |
| Female | 109 | 70 (64%) | 53 (61%) | 17 (77%) | 0.238 |
| Age (years) | 108 | 62 [54-71] | 62 [54-72] | 58 [54-65] | 0.306 |
| ***Medical history*** |  |  |  |  |  |
| Diabetes mellitus | 109 | 69 (63%) | 55 (63%) | 14 (64%) | 0.99 |
| Hypertension | 109 | 62 (57%) | 47 (54%) | 15 (68%) | 0.23 |
| Alcohol abuse | 101 | 24 (23.8%) | 19 (23.2%) | 5 (26.3%) | 0.770 |
| BMI (kg/m²) | 59 | 26.8 [24.3;30.8] | 26.6 [23.0;30.4] | 26.8 [26.0;32.0] | 0.504 |
| Chonic heart failure (NYHA 3-4) | 109 | 15 (14%) | 14 (16%) | 1 (5%) | 0.296 |
| Chronic respiratory failure | 109 | 3 (3%) | 3 (3%) | 0 (0%) | 0.99 |
| COPD | 109 | 4 (4%) | 2 (2%) | 2 (9%) | 0.18 |
| Supraventricular arrhythmia | 109 | 15 (14%) | 12 (14%) | 3 (14%) | 0.99 |
| Liver cirrhosis | 109 | 10 (9%) | 9 (10%) | 1 (5%) | 0.683 |
| Cancer | 109 | 11 (10%) | 10 (12%) | 1 (5%) | 0.457 |
| Hemopathy | 109 | 4 (4%) | 4 (5%) | 0 (0%) | 0.581 |
| Immunodepression | 109 | 19 (17%) | 17 (20%) | 2 (9%) | 0.352 |
| Chronic renal failure | 109 | 24 (22%) | 18 (21%) | 6 (27%) | 0.567 |
| Urinary tract abnormality | 109 | 29 (27%) | 21 (24%) | 8 (36%) | 0.374 |
| Recent NSAID | 108 | 15 (14%) | 11 (13%) | 4 (18%) | 0.502 |
| Delay between hospital admission and ICU admission (days) | 109 | 0.00 [0.00;1.00] | 0.00 [0.00;1.00] | 0.00 [0.00;1.00] | 0.632 |
| Delay between diagnosis and nephrectomy (days) | 19 |  | - | 1 [1-3] |  |
| ***Clinical characteristics upon ICU admission*** |  |  |  |  |  |
| SAPS II at ICU admission | 109 | 51 [37;64] | 44 [36;63] | 60.0 [51;73] | **0.007** |
| SOFA score at ICU admission | 102 | 8.00 [5.00;12.0] | 8.00 [4.00;12.0] | 10.0 [7;14] | **0.018** |
| GCS at ICU admission | 100 | 15 [14;15] | 15 [14;15] | 15 [12;15] | 0.107 |
| Septic shock | 109 | 78 (71.6%) | 58 (67%) | 20 (91%) | **0.047** |
| Bacteriemia | 84 | 59 (70.2%) | 46 (69.7%) | 13 (72.2%) | 0.99 |
| Arterial blood lactate, mmol/L | 94 | 3.50 [2.00;5.70] | 3.46 [1.92;5.07] | 3.85 [2;7] | 0.542 |
| Norepinephrine dose, mg/h | 92 | 1.50 [0.00;4.12] | 1.00 [0.00;3.70] | 4.00 [1;10] | **0.004** |
| Mechanical ventilation | 100 | 41 (41%) | 24 (30%) | 17 (81%) | **<0.001** |
| White cell count, 10^9^ /L | 86 | 13.9 [9.05;19.4] | 14.0 [9.60;19.0] | 13.9 [9;24] | 0.918 |
| Platelet count,10^9^ /L | 98 | 100 [53.2;178] | 104 [60.2;181] | 75.5 [2;162] | 0.122 |
| Natremia, mmol/L | 100 | 134 [128;137] | 133 [128;137] | 134 [126;139] | 0.608 |
| Glycemia , mmol/L | 82 | 10.7 [6.61;18.7] | 10.8 [6.50;18.5] | 10.3 [7.30;19.9] | 0.599 |
| Total bilirubin, μmol/L | 94 | 13.0 [7.;21.8] | 12.0 [7.00;19.8] | 19.0 [14.2;36.8] | **0.017** |
| Serum creatinine, μmol/L | 98 | 260 [152;382] | 256 [145;349] | 317 [237;475] | 0.114 |
| Huang-Tseng scale | 109 |  |  |  | **0.006** |
| 1-2 |  | 65 (60%) | 58 (67%) | 7 (32%) |  |
| 3-4 |  | 44 (40%) | 29 (33%) | 15 (68%) |  |
| ***Treatment at ICU admission*** |  |  |  |  |  |
| Drainage | 109 | 65 (60%) | 55 (63%) | 10 (46%) | 0.203 |
| Combinaison therapy with aminoglycoside | 106 | 84 (79%) | 64 (76%) | 20 (91%) | 0.153 |
| Hydrocortisone | 105 | 16 (15%) | 11 (13%) | 5 (24%) | **0.305** |
| ***Outcome and organ failure*** |  |  |  |  |  |
| ARDS | 105 | 18 (17.1%) | 13 (15.7%) | 5 (22.7%) | 0.525 |
| RRT in ICU | 105 | 33 (31.4%) | 21 (25.0%) | 12 (57.1%) | **0.010** |
| ICU length of stay | 109 | 5.00 [2.00;11.0] | 4.00 [2.00;7.00] | 13.5 [7.25;37.0] | **<0.001** |
| Death in ICU | 109 | 16 (14.7%) | 13 (14.9%) | 3 (13.6%) | 0.99 |
| MAKE 90 | 87 | 35 (40%) | 27 (40%) | 8 (42%) | 0.85 |
| Death at day 90 | 105 | 18 (17%) | 15 (18%) | 3 (14%) | 0.76 |
| Dialysis at day 90* | 87 | 5 (6%) | 2 (3%) | 3 (16%) | 0.07 |
| serum creatinine level ≥1.5 times baseline at day 90 ** | 67 | 21 (31%) | 14 (30%) | 4 (36%) | 0.75 |

ICU: intensive care unit ; SAPS II: Simplified Acute Physiology Score II ; SOFA: Sequential Organ Failure Assessment ; GCS: Glasgow Coma Scale ; NSAID: non-steroidal anti-inflammatory drug ; COPD: chronic obstructive pulmonary disease ; ARDS: acute respiratory distress syndrome; RRT: renal remplacement.*in patients alive at day 90 who were not receiving chronic dialysis before ICU admission ** in patient alive and without dialysis at day 90. Baseline serum creatinine was estimated using the Chronic Kidney Disease–Epidemiology Collaboration (CKD-EPI) equation, assuming an estimated glomerular filtration rate (eGFR) of 75 mL/min/1.73 m² in 25 patients.

**eTable4 Characteristics of patients according to Day-90 mortality (n=105)**

|  | **N Available data** | **Alive at day 90**  **N=87** | **Death at day 90**  **N= 18** | **P value** |
| --- | --- | --- | --- | --- |
| Female | 105 | 57 (66%) | 11 (61%) | 0.932 |
| Age (years) | 104 | 62 [54;70] | 62 [57;76] | 0.338 |
| ***Medical history*** |  |  |  |  |
| Diabetes mellitus | 105 | 58 (67%) | 9 (50%) | 0.285 |
| Hypertension | 105 | 50 (58%) | 9 (50%) | 0.56 |
| Alcohol abuse | 99 | 19 (24%) | 4 (22%) | 0.999 |
| BMI (kg/m²) | 59 | 27.0 [24.2;30.9] | 25.5 [24.6;26.0] | 0.294 |
| Chonic heart failure (NYHA 3-4) | 105 | 11 (13%) | 1 (6%) | 0.686 |
| Chronic respiratory failure | 105 | 2 (2%) | 1 (5%) | 0.435 |
| COPD | 105 | 3 (4%) | 1 (5%) | 0.534 |
| Supraventricular arrhythmia | 105 | 11 (13%) | 2 (11%) | 0.999 |
| Liver cirrhosis | 105 | 5 (6%) | 5 (28%) | **0.013** |
| Cancer | 105 | 5 (6%) | 5 (28%) | **0.013** |
| Hemopathy | 105 | 4 (5%) | 0 (0%) | 0.999 |
| Immunodepression | 105 | 14 (16%) | 4 (22%) | 0.506 |
| Chronic renal failure | 105 | 16 (18%) | 6 (33%) | 0.202 |
| Urinary tract abnormality | 105 | 23 (26%) | 6 (33%) | 0.570 |
| Recent NSAID | 104 | 12 (14%) | 3 (18%) | 0.708 |
| Delay between hospital admission and ICU admission (days) | 105 | 0.00 [0.00;1.00] | 0.00 [0.00;1.00] | 0.710 |
| ***Clinical characteristics upon ICU admission*** |  |  |  |  |
| SAPS II at ICU admission | 105 | 44 [36;58] | 76 [64;86] | **<0.001** |
| SOFA score at ICU admission | 102 | 8 [4;11] | 13 [12;15] | **<0.001** |
| GCS at ICU admission | 98 | 15 [14;15] | 14 [9;15] | **0.006** |
| Septic shock | 105 | 56 (64%) | 18 (100%) | **0.006** |
| Bacteriemia | 82 | 48 (68%) | 10 (91%) | 0.162 |
| Arterial blood lactate, mmol/L | 94 | 3.40 [1.85;4.75] | 8.30 [4.15;12.5] | **0.001** |
| Norepinephrine dose, mg/h | 90 | 1.00 [0.00;3.50] | 11.0 [3.50;15.0] | **<0.001** |
| Mechanical ventilation | 96 | 31 (37%) | 10 (77%) | **0.017** |
| White cell count, 10^9^ /L | 85 | 13.0 [8.85;19.0] | 14.2 [10.2;28.0] | 0.368 |
| Platelet count,10^9^ /L | 98 | 102 [49.0;176] | 79.0 [55.0;202] | 0.851 |
| Natremia, mmol/L | 100 | 134 [128;137] | 132 [126;136] | 0.530 |
| Glycemia, mmol/L | 82 | 10.9 [6.61;18.9] | 9.17 [7.10;13.8] | 0.479 |
| Total bilirubin, μmol/L | 94 | 14.0 [7.00;21.2] | 12.5 [11.0;24.5] | 0.517 |
| Serum creatinine, μmol/L | 96 | 262 [143;374] | 242 [215;368] | 0.917 |
| Huang-Tseng scale | 105 |  |  | 0.304 |
| 1-2 |  | 53 (61%) | 8 (44%) |  |
| 3-4 |  | 34 (39%) | 10 (56%) |  |
| Urinary tract obstruction | 105 | 42 (48%) | 6 (33%) | 0.247 |
| ***Treatment*** |  |  |  |  |
| Drainage | 105 | 51 (60%) | 9 (50% | 0.552 |
| Combinaison therapy with aminoglycoside | 102 | 65 (77%) | 16 (94%) | 0.185 |
| Hydrocortisone | 101 | 10 (12%) | 6 (35%) | 0.027 |
| Nephrectomy | 105 | 19 (22%) | 3 (17%) | 0.759 |
| ***Outcome and organ failure*** |  |  |  |  |
| ARDS | 101 | 10 (12%) | 8 (53%) | **0.001** |
| RRT in ICU | 101 | 25 (29%) | 8 (57%) | 0.062 |

ICU: intensive care unit ; SAPS II: Simplified Acute Physiology Score II ; SOFA: Sequential Organ Failure Assessment ; GCS: Glasgow Coma Scale ; NSAID: non-steroidal anti-inflammatory drug ; COPD: chronic obstructive pulmonary disease ; ARDS: acute respiratory distress syndrome, RRT: renal remplacement.

**eFigure1: Standardized mean differences between groups before and after propensity score weighting and matching.
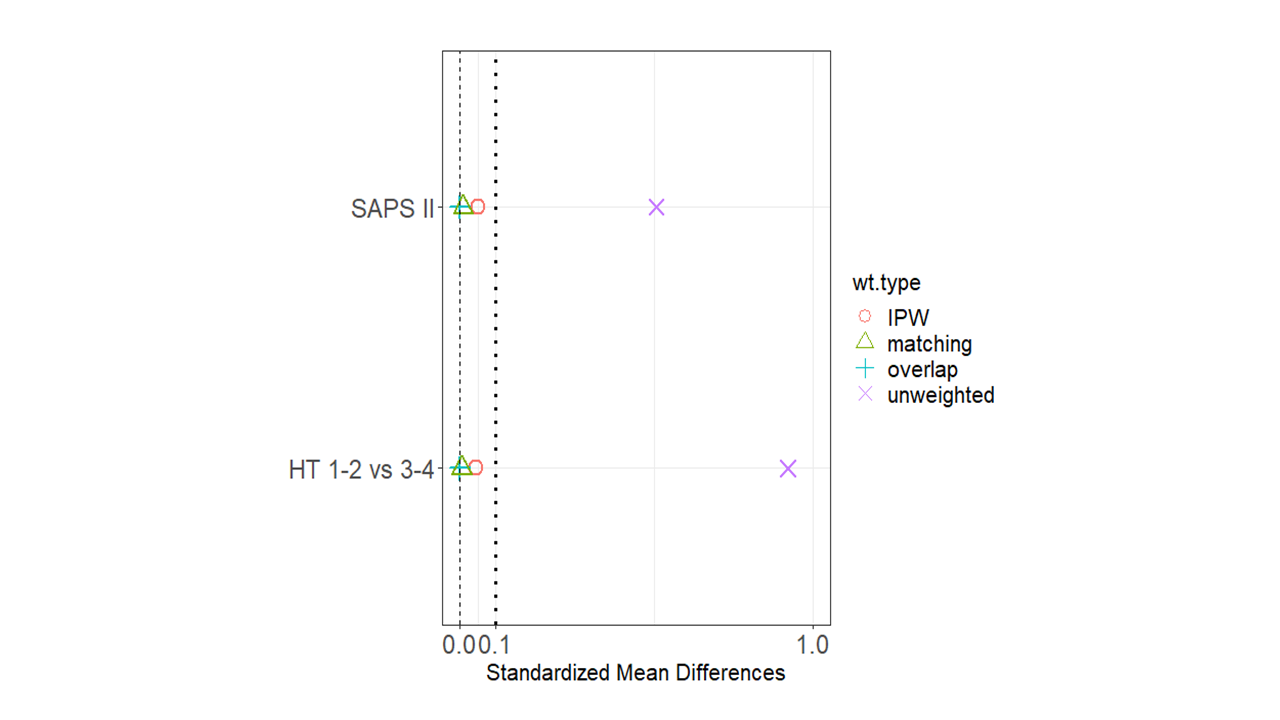
**

**eFigure2: Kaplan–Meier survival curve up to day 90 comparing early nephrectomy (blue line) versus other management strategies (red line).**

**
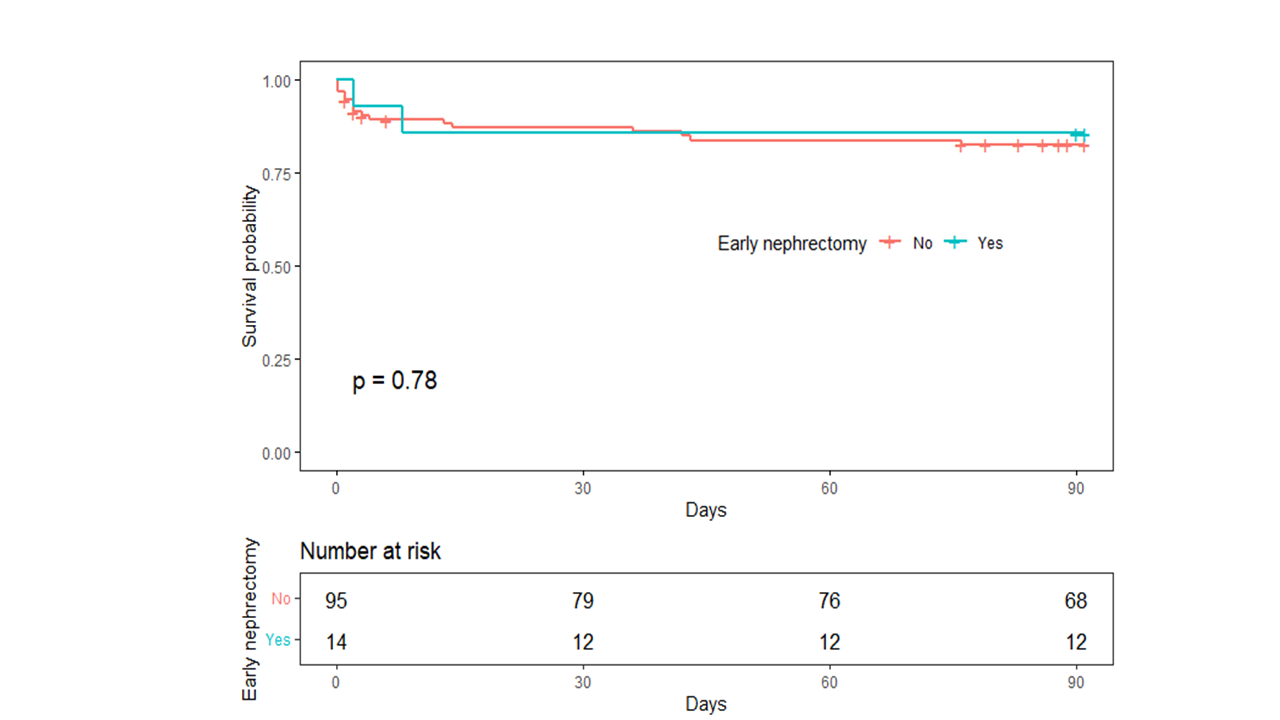
**
